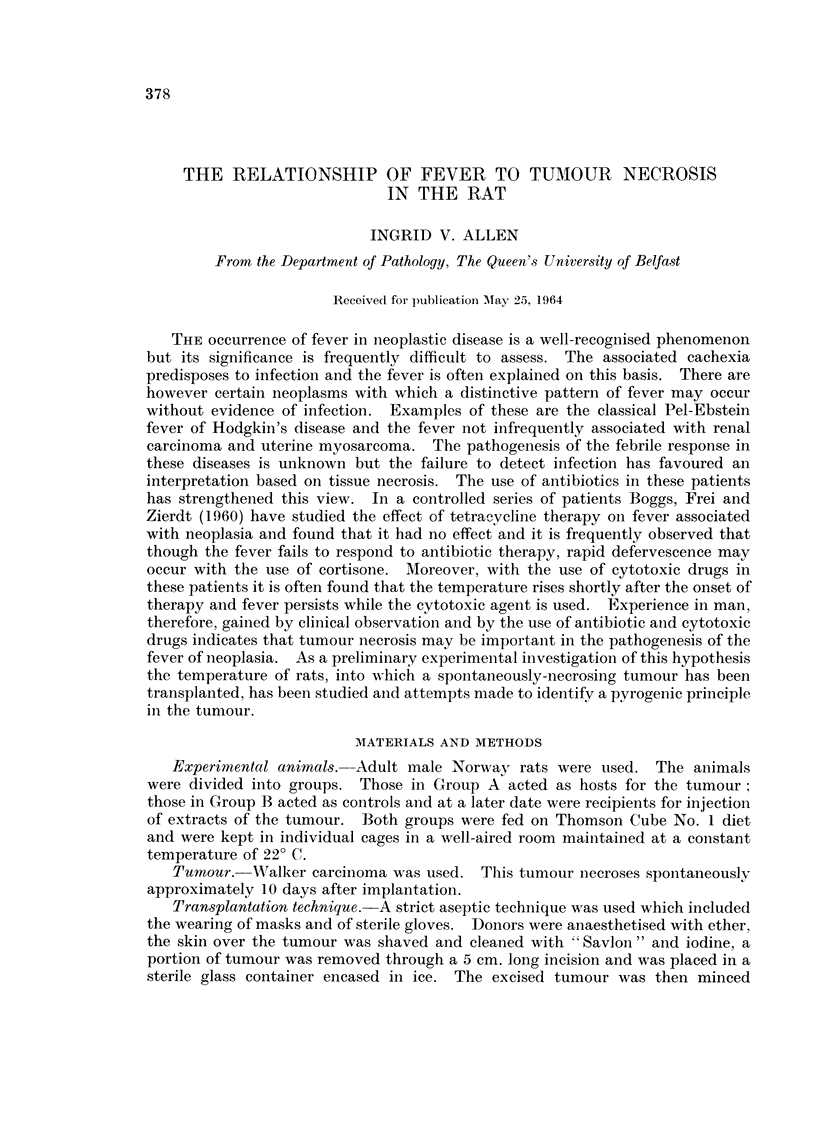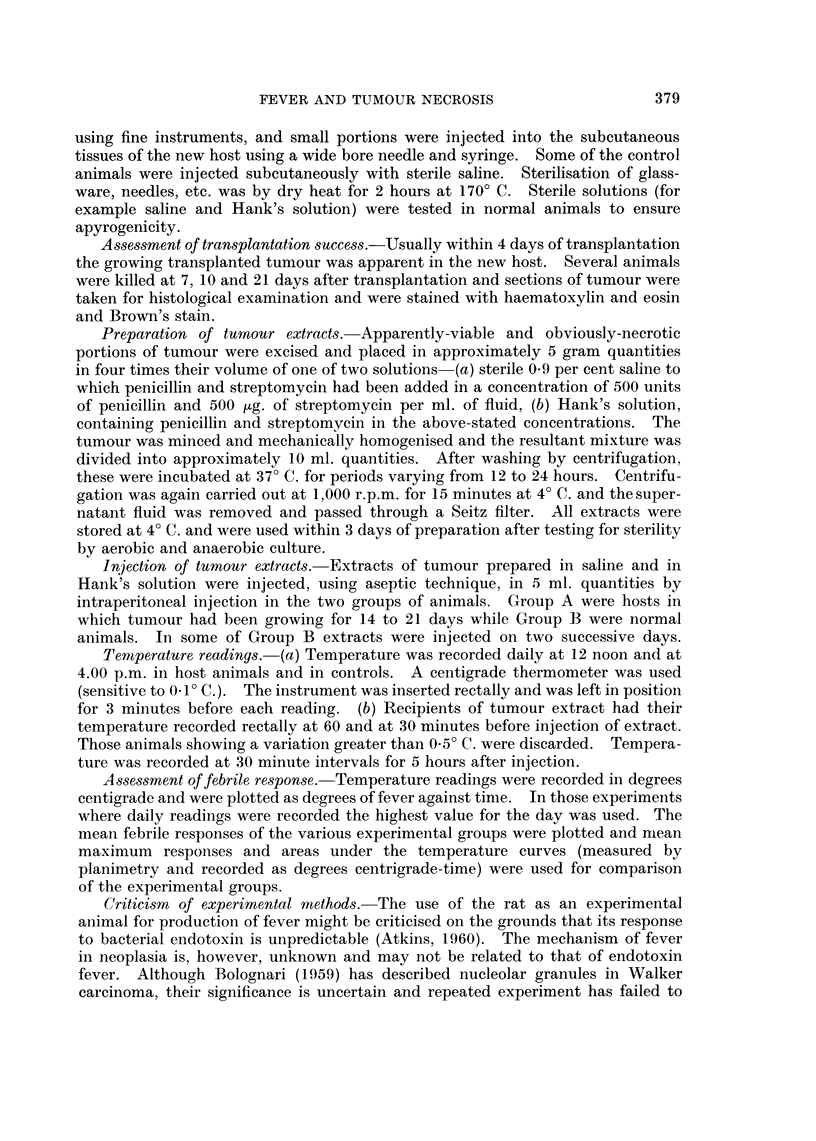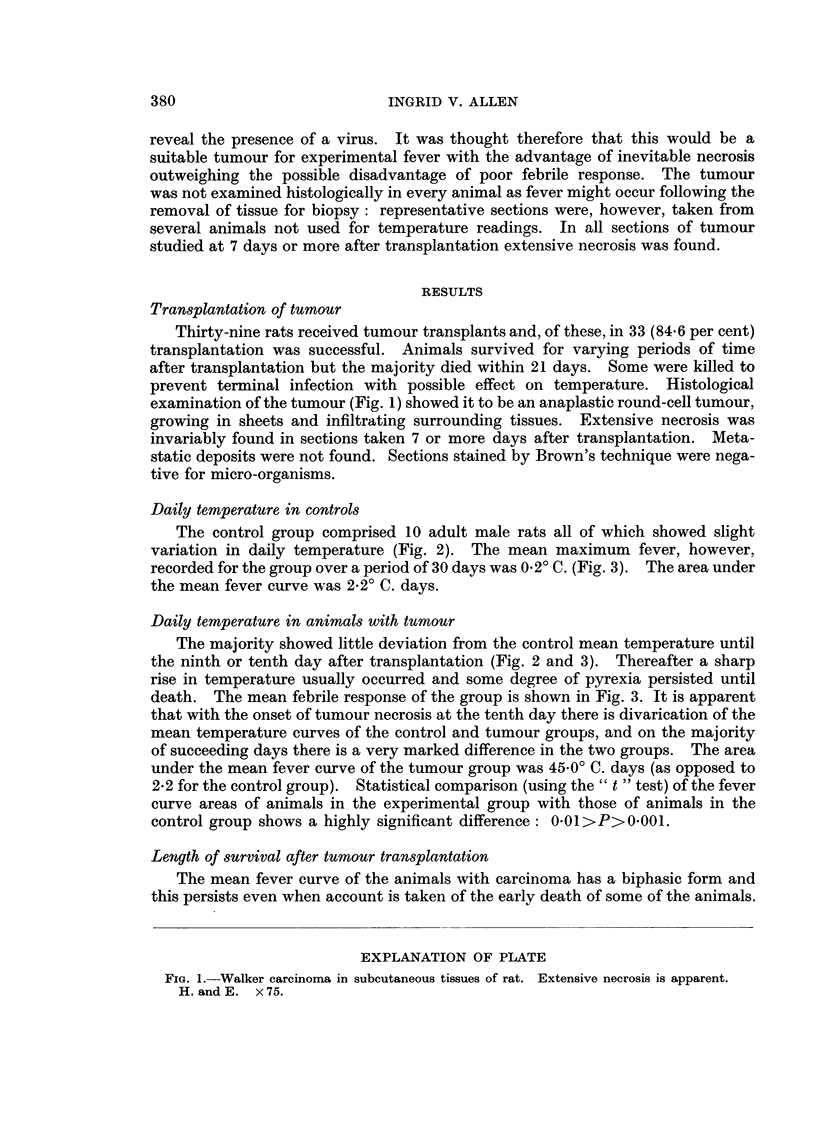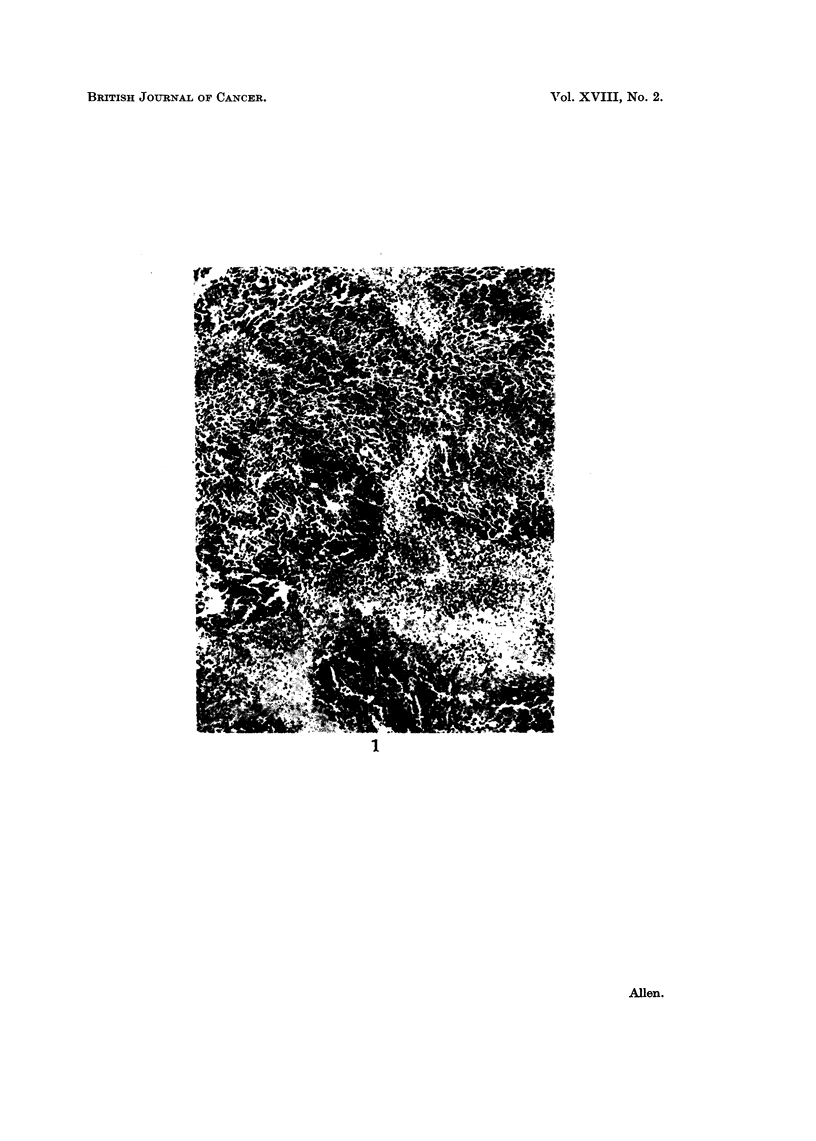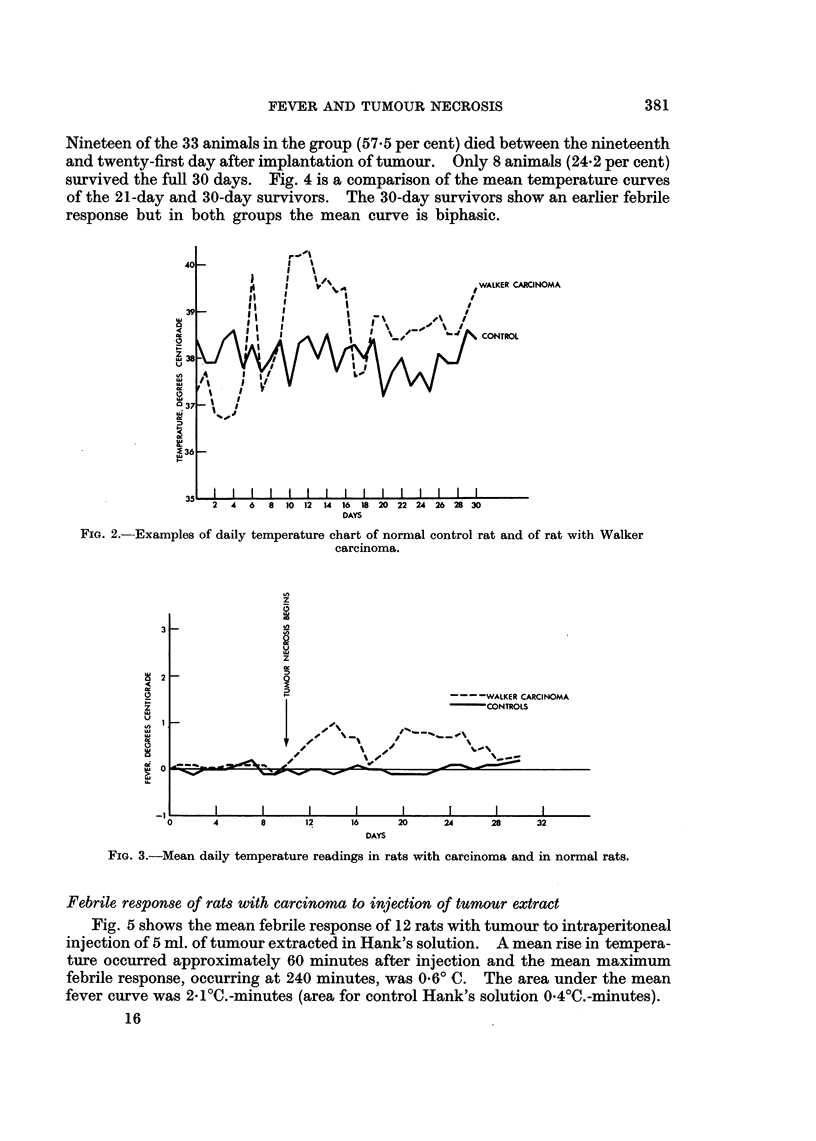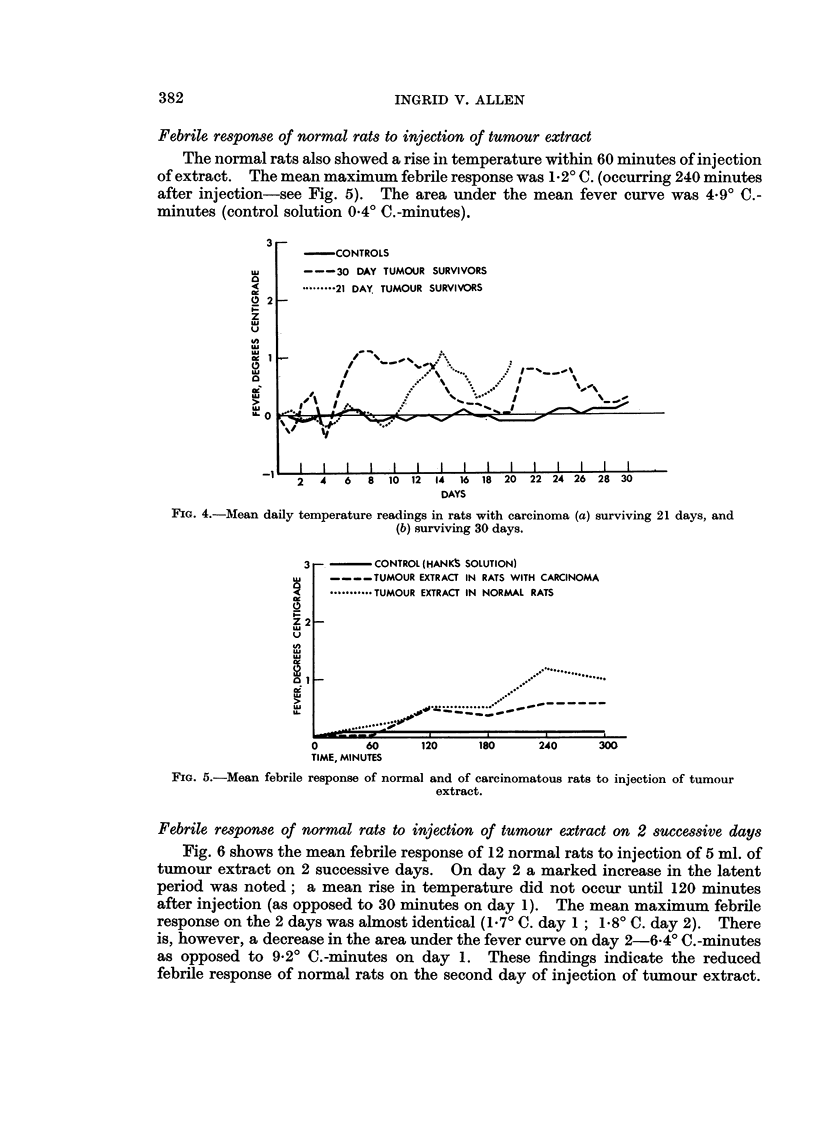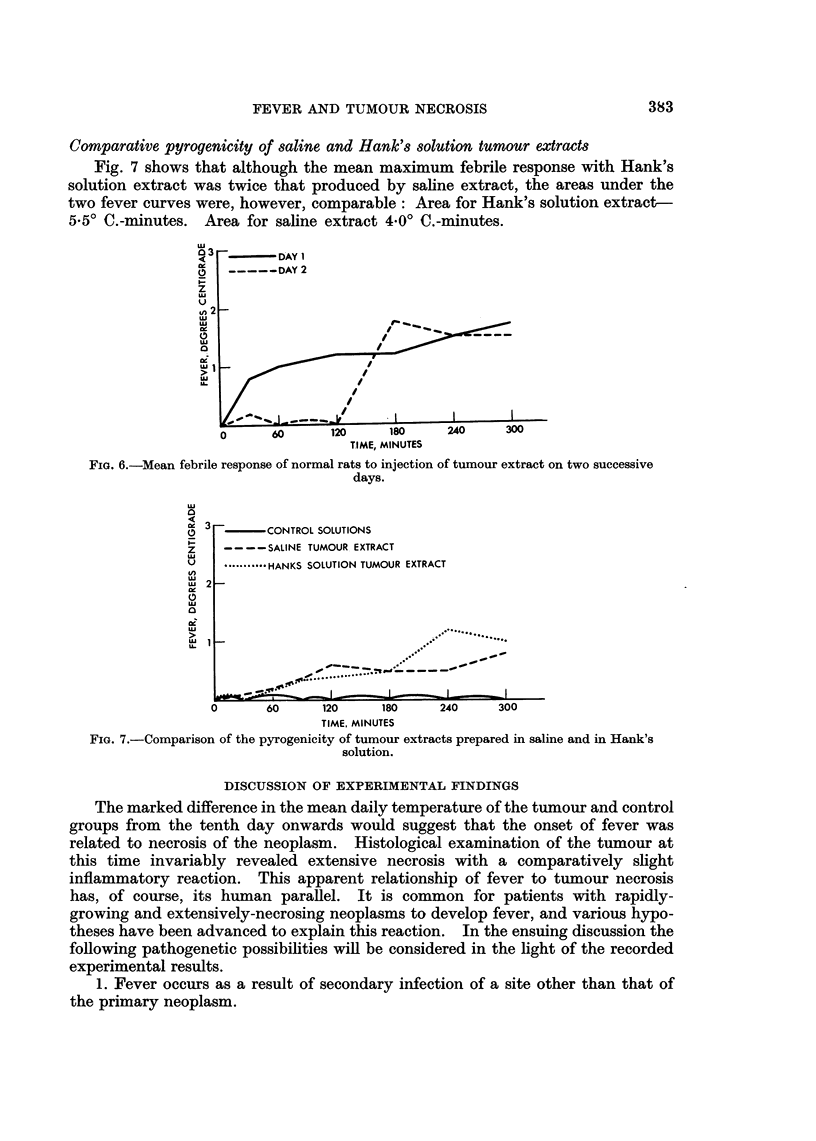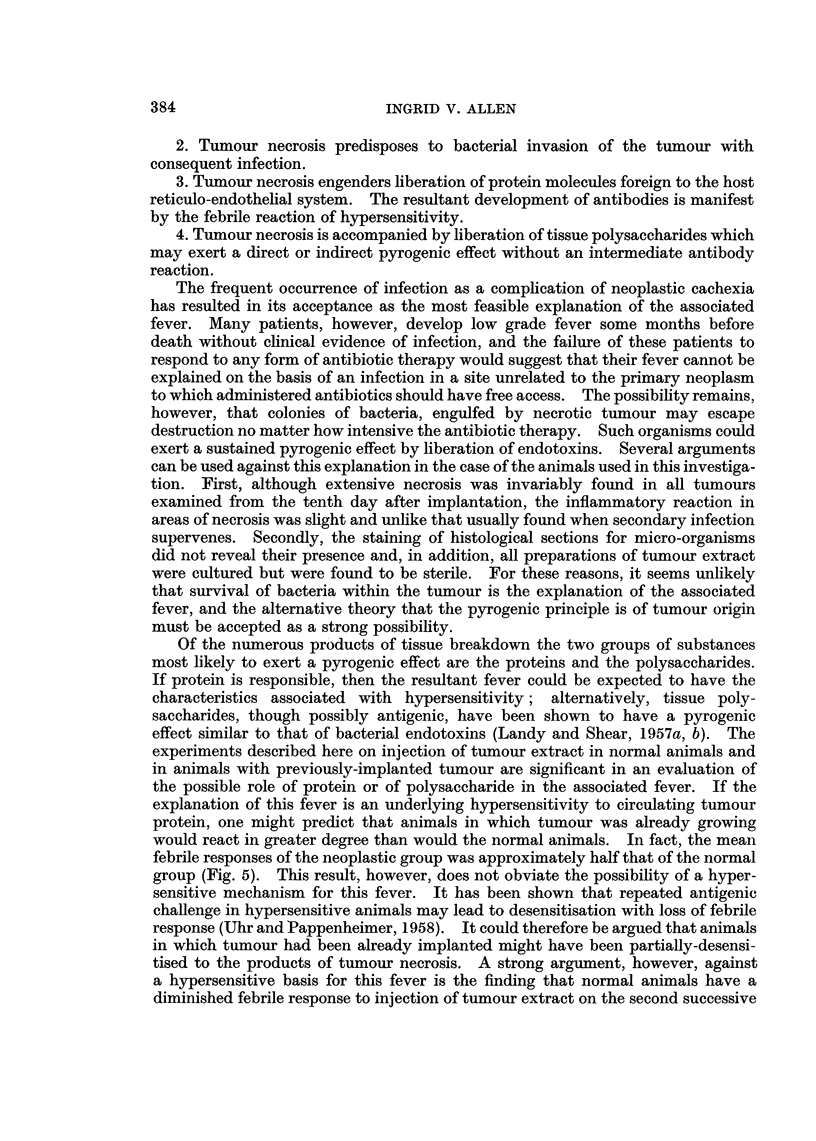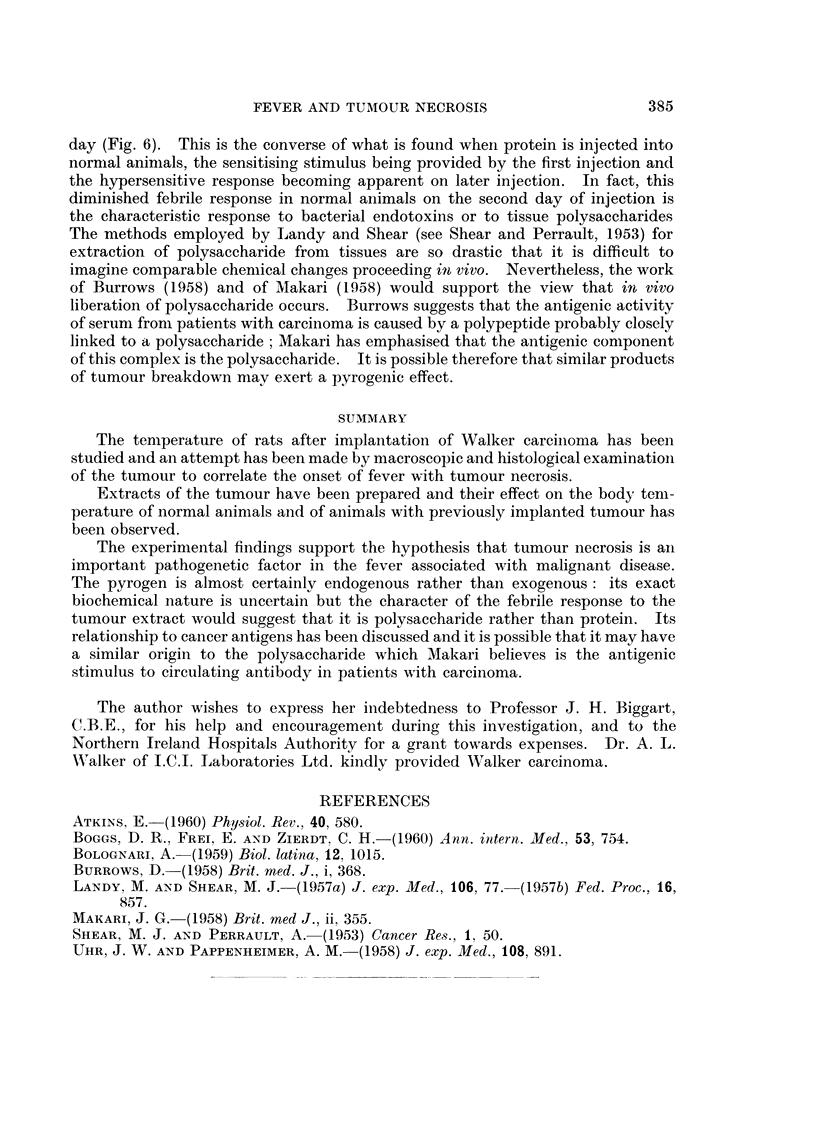# The Relationship of Fever to Tumour Necrosis in the Rat

**DOI:** 10.1038/bjc.1964.43

**Published:** 1964-06

**Authors:** Ingrid V. Allen

## Abstract

**Images:**


### THE RELATIONSHIP OF FEVER TO TUMOUR NECROSIS IN THE RAT

INGRID V. ALLEN

*From the Department of Pathology, The Queen's University of Belfast*

Received for publication May 25, 1964

THE occurrence of fever in neoplastic disease is a well-recognised phenomenon but its significance is frequently difficult to assess. The associated cachexia predisposes to infection and the fever is often explained on this basis. There are however certain neoplasms with which a distinctive pattern of fever may occur without evidence of infection. Examples of these are the classical Pel-Ebstein fever of Hodgkin's disease and the fever not infrequently associated with renal carcinoma and uterine myosarcoma. The pathogenesis of the febrile response in these diseases is unknown but the failure to detect infection has favoured an interpretation based on tissue necrosis. The use of antibiotics in these patients has strengthened this view. In a controlled series of patients Boggs, Frei and Zierdt (1960) have studied the effect of tetracycline therapy on fever associated with neoplasia and found that it had no effect and it is frequently observed that though the fever fails to respond to antibiotic therapy, rapid defervescence may occur with the use of cortisone. Moreover, with the use of cytotoxic drugs in these patients it is often found that the temperature rises shortly after the onset of therapy and fever persists while the cytotoxic agent is used. Experience in man, therefore, gained by clinical observation and by the use of antibiotic and cytotoxic drugs indicates that tumour necrosis may be important in the pathogenesis of the fever of neoplasia. As a preliminary experimental investigation of this hypothesis the temperature of rats, into which a spontaneously-necrosing tumour has been transplanted, has been studied and attempts made to identify a pyrogenic principle in the tumour.

### MATERIALS AND METHODS

*Experimental animals.*—Adult male Norway rats were used. The animals were divided into groups. Those in Group A acted as hosts for the tumour ; those in Group B acted as controls and at a later date were recipients for injection of extracts of the tumour. Both groups were fed on Thomson Cube No. 1 diet and were kept in individual cages in a well-aired room maintained at a constant temperature of 22° C.

*Tumour.*—Walker carcinoma was used. This tumour necroses spontaneously approximately 10 days after implantation.

*Transplantation technique.*—A strict aseptic technique was used which included the wearing of masks and of sterile gloves. Donors were anaesthetised with ether, the skin over the tumour was shaved and cleaned with "Savlon" and iodine, a portion of tumour was removed through a 5 cm. long incision and was placed in a sterile glass container encased in ice. The excised tumour was then minced using fine instruments, and small portions were injected into the subcutaneous tissues of the new host using a wide bore needle and syringe. Some of the control animals were injected subcutaneously with sterile saline. Sterilisation of glassware, needles, etc. was by dry heat for 2 hours at 170° C. Sterile solutions (for example saline and Hank's solution) were tested in normal animals to ensure apyrogenicity.

*Assessment of transplantation success.*—Usually within 4 days of transplantation the growing transplanted tumour was apparent in the new host. Several animals were killed at 7, 10 and 21 days after transplantation and sections of tumour were taken for histological examination and were stained with haematoxylin and eosin and Brown's stain.

*Preparation of tumour extracts.*—Apparently-viable and obviously-necrotic portions of tumour were excised and placed in approximately 5 gram quantities in four times their volume of one of two solutions—(*a*) sterile 0·9 per cent saline to which penicillin and streptomycin had been added in a concentration of 500 units of penicillin and 500 μg. of streptomycin per ml. of fluid, (*b*) Hank's solution, containing penicillin and streptomycin in the above-stated concentrations. The tumour was minced and mechanically homogenised and the resultant mixture was divided into approximately 10 ml. quantities. After washing by centrifugation, these were incubated at 37° C. for periods varying from 12 to 24 hours. Centrifugation was again carried out at 1,000 r.p.m. for 15 minutes at 4° C. and the supernatant fluid was removed and passed through a Seitz filter. All extracts were stored at 4° C. and were used within 3 days of preparation after testing for sterility by aerobic and anaerobic culture.

*Injection of tumour extracts.*—Extracts of tumour prepared in saline and in Hank's solution were injected, using aseptic technique, in 5 ml. quantities by intraperitoneal injection in the two groups of animals. Group A were hosts in which tumour had been growing for 14 to 21 days while Group B were normal animals. In some of Group B extracts were injected on two successive days.

*Temperature readings.*—(*a*) Temperature was recorded daily at 12 noon and at 4.00 p.m. in host animals and in controls. A centigrade thermometer was used (sensitive to 0·1° C.). The instrument was inserted rectally and was left in position for 3 minutes before each reading. (*b*) Recipients of tumour extract had their temperature recorded rectally at 60 and at 30 minutes before injection of extract. Those animals showing a variation greater than 0·5° C. were discarded. Temperature was recorded at 30 minute intervals for 5 hours after injection.

*Assessment of febrile response.*—Temperature readings were recorded in degrees centigrade and were plotted as degrees of fever against time. In those experiments where daily readings were recorded the highest value for the day was used. The mean febrile responses of the various experimental groups were plotted and mean maximum responses and areas under the temperature curves (measured by planimetry and recorded as degrees centrigrade-time) were used for comparison of the experimental groups.

*Criticism of experimental methods.*—The use of the rat as an experimental animal for production of fever might be criticised on the grounds that its response to bacterial endotoxin is unpredictable (Atkins, 1960). The mechanism of fever in neoplasia is, however, unknown and may not be related to that of endotoxin fever. Although Bolognari (1959) has described nucleolar granules in Walker carcinoma, their significance is uncertain and repeated experiment has failed to reveal the presence of a virus. It was thought therefore that this would be a suitable tumour for experimental fever with the advantage of inevitable necrosis outweighing the possible disadvantage of poor febrile response. The tumour was not examined histologically in every animal as fever might occur following the removal of tissue for biopsy : representative sections were, however, taken from several animals not used for temperature readings. In all sections of tumour studied at 7 days or more after transplantation extensive necrosis was found.

### RESULTS

*Transplantation of tumour*

Thirty-nine rats received tumour transplants and, of these, in 33 (84·6 per cent) transplantation was successful. Animals survived for varying periods of time after transplantation but the majority died within 21 days. Some were killed to prevent terminal infection with possible effect on temperature. Histological examination of the tumour (Fig. 1) showed it to be an anaplastic round-cell tumour, growing in sheets and infiltrating surrounding tissues. Extensive necrosis was invariably found in sections taken 7 or more days after transplantation. Metastatic deposits were not found. Sections stained by Brown's technique were negative for micro-organisms.

*Daily temperature in controls*

The control group comprised 10 adult male rats all of which showed slight variation in daily temperature (Fig. 2). The mean maximum fever, however, recorded for the group over a period of 30 days was 0·2° C. (Fig. 3). The area under the mean fever curve was 2·2° C. days.

*Daily temperature in animals with tumour*

The majority showed little deviation from the control mean temperature until the ninth or tenth day after transplantation (Fig. 2 and 3). Thereafter a sharp rise in temperature usually occurred and some degree of pyrexia persisted until death. The mean febrile response of the group is shown in Fig. 3. It is apparent that with the onset of tumour necrosis at the tenth day there is divarication of the mean temperature curves of the control and tumour groups, and on the majority of succeeding days there is a very marked difference in the two groups. The area under the mean fever curve of the tumour group was 45·0° C. days (as opposed to 2·2 for the control group). Statistical comparison (using the " $t$ " test) of the fever curve areas of animals in the experimental group with those of animals in the control group shows a highly significant difference : $0{\cdot}01 > P > 0{\cdot}001$.

*Length of survival after tumour transplantation*

The mean fever curve of the animals with carcinoma has a biphasic form and this persists even when account is taken of the early death of some of the animals.

---

EXPLANATION OF PLATE

FIG. 1.—Walker carcinoma in subcutaneous tissues of rat. Extensive necrosis is apparent. H. and E. ×75.

1

Allen.

Nineteen of the 33 animals in the group (57·5 per cent) died between the nineteenth and twenty-first day after implantation of tumour. Only 8 animals (24·2 per cent) survived the full 30 days. Fig. 4 is a comparison of the mean temperature curves of the 21-day and 30-day survivors. The 30-day survivors show an earlier febrile response but in both groups the mean curve is biphasic.

FIG. 2.—Examples of daily temperature chart of normal control rat and of rat with Walker carcinoma.

FIG. 3.—Mean daily temperature readings in rats with carcinoma and in normal rats.

*Febrile response of rats with carcinoma to injection of tumour extract*

Fig. 5 shows the mean febrile response of 12 rats with tumour to intraperitoneal injection of 5 ml. of tumour extracted in Hank's solution. A mean rise in temperature occurred approximately 60 minutes after injection and the mean maximum febrile response, occurring at 240 minutes, was 0·6° C. The area under the mean fever curve was 2·1°C.-minutes (area for control Hank's solution 0·4°C.-minutes).

*Febrile response of normal rats to injection of tumour extract*

The normal rats also showed a rise in temperature within 60 minutes of injection of extract. The mean maximum febrile response was 1·2° C. (occurring 240 minutes after injection—see Fig. 5). The area under the mean fever curve was 4·9° C.-minutes (control solution 0·4° C.-minutes).

FIG. 4.—Mean daily temperature readings in rats with carcinoma (*a*) surviving 21 days, and (*b*) surviving 30 days.

FIG. 5.—Mean febrile response of normal and of carcinomatous rats to injection of tumour extract.

*Febrile response of normal rats to injection of tumour extract on 2 successive days*

Fig. 6 shows the mean febrile response of 12 normal rats to injection of 5 ml. of tumour extract on 2 successive days. On day 2 a marked increase in the latent period was noted ; a mean rise in temperature did not occur until 120 minutes after injection (as opposed to 30 minutes on day 1). The mean maximum febrile response on the 2 days was almost identical (1·7° C. day 1 ; 1·8° C. day 2). There is, however, a decrease in the area under the fever curve on day 2—6·4° C.-minutes as opposed to 9·2° C.-minutes on day 1. These findings indicate the reduced febrile response of normal rats on the second day of injection of tumour extract.

*Comparative pyrogenicity of saline and Hank's solution tumour extracts*

Fig. 7 shows that although the mean maximum febrile response with Hank's solution extract was twice that produced by saline extract, the areas under the two fever curves were, however, comparable : Area for Hank's solution extract—5·5° C.-minutes. Area for saline extract 4·0° C.-minutes.

FIG. 6.—Mean febrile response of normal rats to injection of tumour extract on two successive days.

FIG. 7.—Comparison of the pyrogenicity of tumour extracts prepared in saline and in Hank's solution.

### DISCUSSION OF EXPERIMENTAL FINDINGS

The marked difference in the mean daily temperature of the tumour and control groups from the tenth day onwards would suggest that the onset of fever was related to necrosis of the neoplasm. Histological examination of the tumour at this time invariably revealed extensive necrosis with a comparatively slight inflammatory reaction. This apparent relationship of fever to tumour necrosis has, of course, its human parallel. It is common for patients with rapidly-growing and extensively-necrosing neoplasms to develop fever, and various hypotheses have been advanced to explain this reaction. In the ensuing discussion the following pathogenetic possibilities will be considered in the light of the recorded experimental results.

1. Fever occurs as a result of secondary infection of a site other than that of the primary neoplasm.

2. Tumour necrosis predisposes to bacterial invasion of the tumour with consequent infection.

3. Tumour necrosis engenders liberation of protein molecules foreign to the host reticulo-endothelial system. The resultant development of antibodies is manifest by the febrile reaction of hypersensitivity.

4. Tumour necrosis is accompanied by liberation of tissue polysaccharides which may exert a direct or indirect pyrogenic effect without an intermediate antibody reaction.

The frequent occurrence of infection as a complication of neoplastic cachexia has resulted in its acceptance as the most feasible explanation of the associated fever. Many patients, however, develop low grade fever some months before death without clinical evidence of infection, and the failure of these patients to respond to any form of antibiotic therapy would suggest that their fever cannot be explained on the basis of an infection in a site unrelated to the primary neoplasm to which administered antibiotics should have free access. The possibility remains, however, that colonies of bacteria, engulfed by necrotic tumour may escape destruction no matter how intensive the antibiotic therapy. Such organisms could exert a sustained pyrogenic effect by liberation of endotoxins. Several arguments can be used against this explanation in the case of the animals used in this investigation. First, although extensive necrosis was invariably found in all tumours examined from the tenth day after implantation, the inflammatory reaction in areas of necrosis was slight and unlike that usually found when secondary infection supervenes. Secondly, the staining of histological sections for micro-organisms did not reveal their presence and, in addition, all preparations of tumour extract were cultured but were found to be sterile. For these reasons, it seems unlikely that survival of bacteria within the tumour is the explanation of the associated fever, and the alternative theory that the pyrogenic principle is of tumour origin must be accepted as a strong possibility.

Of the numerous products of tissue breakdown the two groups of substances most likely to exert a pyrogenic effect are the proteins and the polysaccharides. If protein is responsible, then the resultant fever could be expected to have the characteristics associated with hypersensitivity; alternatively, tissue polysaccharides, though possibly antigenic, have been shown to have a pyrogenic effect similar to that of bacterial endotoxins (Landy and Shear, 1957*a*, *b*). The experiments described here on injection of tumour extract in normal animals and in animals with previously-implanted tumour are significant in an evaluation of the possible role of protein or of polysaccharide in the associated fever. If the explanation of this fever is an underlying hypersensitivity to circulating tumour protein, one might predict that animals in which tumour was already growing would react in greater degree than would the normal animals. In fact, the mean febrile responses of the neoplastic group was approximately half that of the normal group (Fig. 5). This result, however, does not obviate the possibility of a hypersensitive mechanism for this fever. It has been shown that repeated antigenic challenge in hypersensitive animals may lead to desensitisation with loss of febrile response (Uhr and Pappenheimer, 1958). It could therefore be argued that animals in which tumour had been already implanted might have been partially-desensitised to the products of tumour necrosis. A strong argument, however, against a hypersensitive basis for this fever is the finding that normal animals have a diminished febrile response to injection of tumour extract on the second successive day (Fig. 6). This is the converse of what is found when protein is injected into normal animals, the sensitising stimulus being provided by the first injection and the hypersensitive response becoming apparent on later injection. In fact, this diminished febrile response in normal animals on the second day of injection is the characteristic response to bacterial endotoxins or to tissue polysaccharides The methods employed by Landy and Shear (see Shear and Perrault, 1953) for extraction of polysaccharide from tissues are so drastic that it is difficult to imagine comparable chemical changes proceeding *in vivo*. Nevertheless, the work of Burrows (1958) and of Makari (1958) would support the view that *in vivo* liberation of polysaccharide occurs. Burrows suggests that the antigenic activity of serum from patients with carcinoma is caused by a polypeptide probably closely linked to a polysaccharide ; Makari has emphasised that the antigenic component of this complex is the polysaccharide. It is possible therefore that similar products of tumour breakdown may exert a pyrogenic effect.

### SUMMARY

The temperature of rats after implantation of Walker carcinoma has been studied and an attempt has been made by macroscopic and histological examination of the tumour to correlate the onset of fever with tumour necrosis.

Extracts of the tumour have been prepared and their effect on the body temperature of normal animals and of animals with previously implanted tumour has been observed.

The experimental findings support the hypothesis that tumour necrosis is an important pathogenetic factor in the fever associated with malignant disease. The pyrogen is almost certainly endogenous rather than exogenous : its exact biochemical nature is uncertain but the character of the febrile response to the tumour extract would suggest that it is polysaccharide rather than protein. Its relationship to cancer antigens has been discussed and it is possible that it may have a similar origin to the polysaccharide which Makari believes is the antigenic stimulus to circulating antibody in patients with carcinoma.

The author wishes to express her indebtedness to Professor J. H. Biggart, C.B.E., for his help and encouragement during this investigation, and to the Northern Ireland Hospitals Authority for a grant towards expenses. Dr. A. L. Walker of I.C.I. Laboratories Ltd. kindly provided Walker carcinoma.

### REFERENCES

ATKINS, E.—(1960) *Physiol. Rev.*, **40**, 580.
BOGGS, D. R., FREI, E. AND ZIERDT, C. H.—(1960) *Ann. intern. Med.*, **53**, 754.
BOLOGNARI, A.—(1959) *Biol. latina*, **12**, 1015.
BURROWS, D.—(1958) *Brit. med. J.*, i, 368.
LANDY, M. AND SHEAR, M. J.—(1957*a*) *J. exp. Med.*, **106**, 77.—(1957*b*) *Fed. Proc.*, **16**, 857.
MAKARI, J. G.—(1958) *Brit. med J.*, ii, 355.
SHEAR, M. J. AND PERRAULT, A.—(1953) *Cancer Res.*, **1**, 50.
UHR, J. W. AND PAPPENHEIMER, A. M.—(1958) *J. exp. Med.*, **108**, 891.